# Factors Affecting Patulin Production by *Penicillium expansum* in Apples

**DOI:** 10.3390/foods14132310

**Published:** 2025-06-30

**Authors:** Tamara Edina Gal, Ersilia Călina Alexa, Renata Maria Șumălan, Ionuț Dascălu, Olimpia Alina Iordănescu

**Affiliations:** 1 Department of Horticulture, Faculty of Engineering and Applied Technologies, University of Life Sciences “King Mihai I” from Timișoara, 300645 Timișoara, Romania; tamara-edina.gal.fita@usvt.ro (T.E.G.); olimpiaiordanescu@usvt.ro (O.A.I.); 2 Department of Food Control, Faculty of Food Engineering, University of Life Sciences “King Mihai I” from Timișoara, 300645 Timișoara, Romania; ersiliaalexa@usvt.ro; 3 Department of Silviculture, Faculty of Engineering and Applied Technologies, University of Life Sciences “King Mihai I” from Timișoara, 300645 Timișoara, Romania; renatasumalan@usvt.ro

**Keywords:** PAT, *Penicillium expansum*, apple, postharvest, blue mold

## Abstract

The main mycotoxin found in apples is patulin (PAT), mostly produced by *Penicillium expansum*, during the storage of fruits. It is very difficult to control the quality of every fruit that enters the processing line, so there is a high probability that apple juice, applesauce, apple cider, even products intended for babies, contain moldy fruits, with PAT content. This review paper provides detailed information about the extrinsic and intrinsic factors that affect PAT prevalence in apples. Extrinsic factors, such as temperature, air composition in the storage room or packaging material, play a key role in infection with *P. expansum* and PAT accumulation. Lower temperatures often prevent fungal growth and the production of the mycotoxin, whereas higher or unstable temperatures can promote the buildup of the toxin in infected fruits. Controlled atmosphere storage appears to inhibit the accumulation of PAT in apples. In terms of internal composition, variations in the pH of the fruits and flesh firmness significantly impact fungal growth and PAT production in the fruits. The presence of ethylene, sucrose and polyphenols are some of the decisive chemical components that regulate PAT buildup. Susceptibility of different cultivars is also genetically driven, but the size of the decay area and the toxin-producing capacity of the fungal strain have noteworthy influence as well. Knowledge of these elements helps to understand the mechanisms of PAT production.

## 1. Introduction

Fruits’ postharvest deterioration is a major contributor to postharvest losses. Most fruits are perishable crops, as during storage period, a number of diseases caused by microorganisms are likely to grow and develop in them because of their increased moisture content (roughly 70% to 95% water), high respiration rate, and mostly soft texture [1,2]. The infection occurs in the orchard (from the soil or the air) but remains in a latent state until the fruit reaches maturity. Improper handling and injury to the fruit before, during or after harvest can lead to further development of the disease and often compromises a high percentage of the yields [3,4,5]. Fungal spoilage causes nutrient depletion and aesthetic problems in fruits and is the primary source of financial losses in the postharvest phase [6]. Fruits contaminated with fungal infections also raise the risk of consumers developing serious health problems, as fungi are able to produce toxic secondary metabolites, named mycotoxins [7,8].

Apple (*Malus domestica* Borkh.) is the biggest fruit crop grown in temperate climates and one of the most widely consumed fruit in the world [9]. Apples are beneficial to human health and nutrition due to their rich biochemical composition [10,11]. Another quality of this temperate fruit is its extended shelf life, which guarantees a steady supply of fresh fruits throughout the year [12], but fungal diseases during storage are responsible for roughly 25–50% of apple product losses worldwide [13]. Apples can be stored for a few months on average, in cold environment and modified atmosphere this period can be extended even more [14,15]. Apple genotype, management practices before and after harvest, environmental factors all influence the probability of spoilage [16]. Additionally, apples’ nutritional constitution is advantageous for the growth of harmful fungi [17]. *Penicillium expansum (P. expansum)*, *Botrytis cinerea*, *Botryosphaeria dothidea*, *Monilia* spp., *Alternaria* spp., *Byssochlamys* spp., *Fusarium* spp., *Aspergillus* spp. are among the fungal species that often affect apples [18,19,20,21].

While certain postharvest microorganisms have the ability to directly infect fruits, *P. expansum* does not, since it penetrates through natural openings (lenticels, calyx) or wounds that occur prior to, during or postharvest, frequently as a result of severe bruising and stem punctures [4,22]. Therefore fruits that suffered physical damage during handling are more likely to become infected with the fungus later on [23].

**Figure 1:**
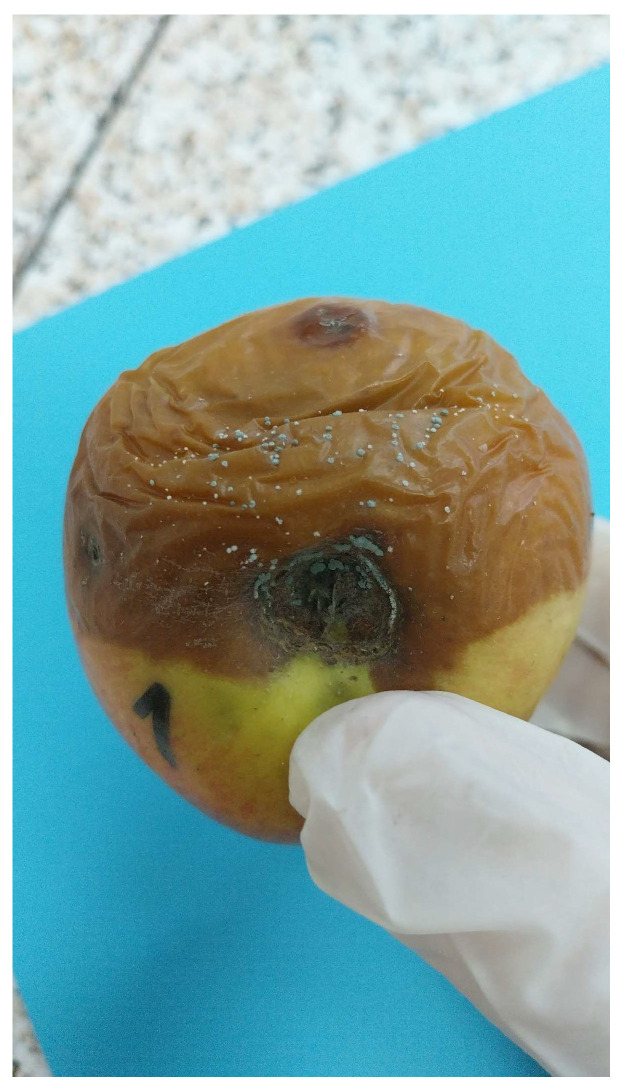
Apple with typical symptoms of *P. expansum* infection, showing soft, watery, brown lesion and visible blue-green conidia (original photo).

*P. expansum* is a filamentous fungus widespread in the environment that can cause blue mold, which is one of the most prevalent postharvest diseases of apples [24]. Its name refers to the color of the conidia produced by the fungi. The first symptoms of the infection are the soft, watery, light brown lesions. As the lesions become older, the conidia become blue-green (Figure 1). The disease is frequently called ‘soft rot’, referring to the watery, soft texture of the rotten tissue. Healthy tissue can be easily separated from decayed one. It is of great importance to note, however, that the mycotoxin produced in apples by this fungus, namely patulin (PAT), can migrate within the fruit, so the absence of the mold from healthy tissue does not necessarily mean that it does not contain PAT [25]. As mentioned earlier, blue-green spores and conidial tufts of *P. expansum* may cover the surface of aged lesions. Rotten fruits have a musty, earthy smell. This odor and the blue-green spores are the obvious indicators of the disease [5,26]. In general, the symptoms do not become visible until the fruits have been stored for a few weeks [27]. Sometimes the spoilage is not visible from the outside, but internal spoilage can cause PAT production as well [28].

*P. expansum* is the primary PAT-producing fungus [29], which means that infection with this species might determine the accumulation of PAT as well [30,31], causing severe health issues [6].

**Figure 2:**
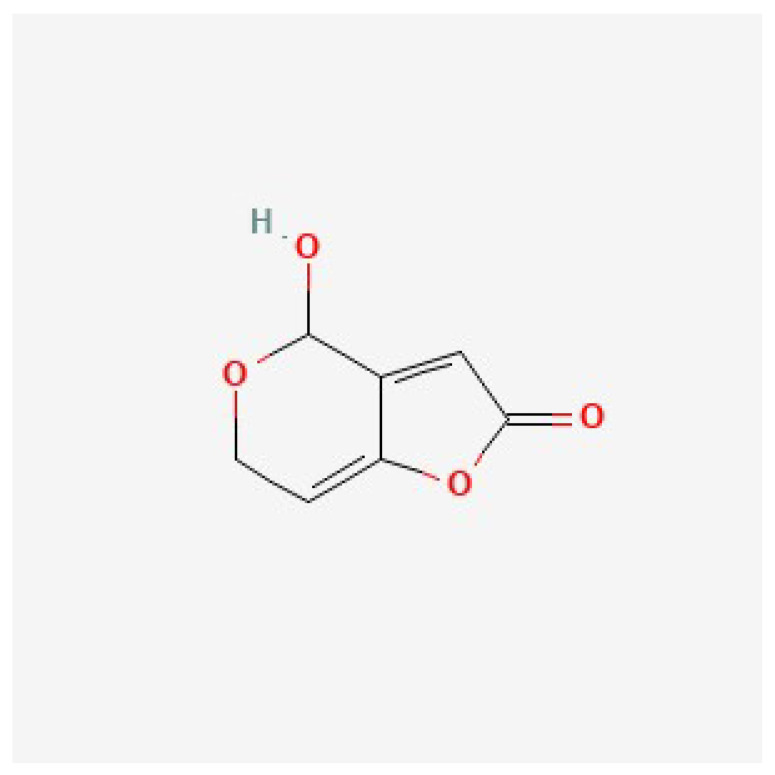
Chemical structure of patulin (C_7_H_6_O_4_) (source: National Center for Biotechnology Information. Available online: https://pubchem.ncbi.nlm.nih.gov/compound/Patulin (accessed on 25 June 2025).

PAT (4-hydroxy-4H-furo [3,2c] pyran-2[6H]-one) (chemical structure in Figure 2) is one of the most concerning mycotoxins, produced specifically by *Penicillium*, *Aspergillus* and *Byssochlamys* species [32,33]. Fruits, vegetables, cereals are among the foods that have been shown to contain PAT [34], nonetheless, it is mostly present in apples and its derivatives [35]. Due to its stable chemical characteristics, PAT can withstand high temperatures during processing [36,37]. PAT can cause neurotoxicity, genotoxicity and immunotoxicity, as well as different health conditions of the liver, kidneys, lungs, the gastrointestinal tract [32,38,39,40].

According to the European Commission, the highest permitted amounts of PAT in apple juice and cider are 50 µg/L, in solid apple products 25 µg/kg, and in products intended for consumption by babies and young children 10 µg/kg [41]. The acceptable level for babies is set at 5 times lower than that for adults. In addition to being more exposed per kilogram of body weight, they are more vulnerable because of their unique physiology as well [42]. Pregnant women are also considered as high risk population [43].

Apples in various processed forms are consumed in fairly high quantities by all age groups. Given the frequency with which this storage disease occurs and the serious consequences of chronic exposure to PAT, it is of paramount importance that apples used in industrialization are sound, free of mold.

## 2. External and Inherent Components That Drive Patulin Biosynthesis

### 2.1. Extrinsic Components That Influence Patulin Production

#### 2.1.1. Storage Temperature

*P. expansum*’s PAT production is greatly influenced by storage temperature. Lower temperatures often prevent fungal growth and the production of the mycotoxin, whereas higher or unstable temperatures can promote the buildup of the toxin in infected fruits.

The optimum temperatures for the development of *P. expansum* spores and mycelia are between 20 and 25 °C [44,45].

Salomão et al. [46] studied the effect of variety and storage temperature on PAT production in ‘*Red Delicious*’, ‘*Golden Supreme*’, ‘*Gala*’, ‘*Fuji*’, ‘*Empire*’ and ‘*McIntosh*’ apple varieties, artificially inoculated with *P. expansum* spore suspension. Although both storage temperatures (20.5 °C and 11 °C) resulted in the production of PAT, apples incubated at 20.5 °C produced much more PAT than apples incubated at 11 °C. 44% of the examined samples had PAT concentrations higher than the 50 ppb regulation limit set by the US Food and Drug Administration. The author’s observations indicate that higher temperatures stimulate PAT production, while lower temperatures can have limiting effect on PAT synthesis in rotten apples during the storage period. This is in agreement with other studies in the field [47,48,49,50,51,52].

However, sometimes refrigeration temperatures can enhance PAT production [53]. When the temperature was decreased from 20 °C to 10 and 4 °C, PAT synthesis was stimulated. Only an even stronger drop, to 1 °C, caused a reduction in PAT levels. However, this trend was not observed in all strains, which proves that PAT production is also strongly strain-dependent. In the same study, the influence of temperature fluctuations on fungal growth was also monitored. At the optimal temperature (25 °C), the impact of a slight temperature change was less noticeable on fungal growth than at low temperatures (2 °C, 4 °C). While the lag phase at 25 °C and 20 °C differed by only 24 h (24 and 48 h, respectively), at 2 °C it was 150 h longer than at 4 °C (400 and 250 h, respectively). For growth rate, a similar tendency was noticed.

Several studies correlated storage temperature and duration of storage when measuring PAT accumulation in apples. PAT quantities were higher after a shorter time period but with high temperature, compared to longer storage periods at low temperatures: 538–1822 mg/mL PAT, day 14 at 25 °C and 75–396 mg/mL PAT, day 24 at 4 °C [54]; 800–12,500 µg PAT/kg rotten tissue, 9 days at 25 °C and 800–1200 µg PAT/kg rotten tissue, 45 days at 4 °C [50]; Welke et al. found that the longest duration of time that apples could be stored at 4 °C, without developing PAT, was 27 days. Both the diameter of the lesion and PAT accumulation considerably increased when the fruits were stored at 25 °C for 3 days [55].

A strain-specific adjustment to stress is suggested by Garcia et al. [56], as isolates that produced higher amounts of PAT at 20 °C generally differed from the strains that produced more at 1 °C.

Despite the fact that there are some contradictions throughout the existing studies, it can be concluded that, in general, lower temperatures result in less PAT synthesis than higher temperatures.

#### 2.1.2. Atmospheric Regimes and Packaging Materials Used During Controlled Atmosphere (CA) Storage

Apples are typically harvested in the pre-climacteric (unripened) or climacteric (commercially ripened) stages and are often sent to CA storage chambers, which may help delay ripening and increase shelf-life so that fresh fruit is available all year round [57].

Ripening of fruits and PAT biosynthesis are significantly impacted by the gas composition in the storage room, especially O_2_ and CO_2_ levels, and by the materials used for the packaging of the fruits. Their combined application is encouraged in order to increase their efficacy in maintaining the fruits’ quality and freshness, storability and their microbiological safety.

Morales et al. [47] revealed that apples kept cold for 60 days under CA circumstances had lesions that were 2–4 cm in diameter, but no PAT was found.

The composition of the air impacts flesh firmness, which influences the fruit’s reaction to pathogen infection and PAT production. The direct effect of limited oxygen on PAT synthesis is questionable though [53,58,59]. There are studies, however, that describe that extremely low oxygen levels during storage contributed to lower PAT accumulation due to delayed fruit ripening [60]. Although the effect of low O_2_ concentrations (3%) on PAT accumulation varies per strain, Baert et al. (2007) discovered that 1% O_2_ in the atmosphere clearly reduces PAT accumulation regardless of the strain under investigation [53]. The authors came to the conclusion that in order to inhibit the formation of PAT, the temperature and partial pressure of oxygen should be as low as possible. It appears that *P. expansum*’s primary and secondary metabolisms are reduced under these stressors (low temperatures, low O_2_, and high CO_2_).

A total of 100 mL of *P. expansum* spore suspension was injected into healthy ‘*Granny Smith*’ apples to a depth of 1 cm, at two distinct points. In different package materials, exposed to various atmospheric regimes (three CO_2_/N_2_ mixes: 48/52%, 58/42%, 88/12% and atmospheric gas), the fruits were incubated for 14 days at 25 °C. Polypropylene (PP) and polyethylene (PE) packaging materials were used, as well as control samples (without packaging). Fungal development was tracked at each two to four days, and after 14 days high-performance liquid chromatography (HPLC) was used to measure the PAT content of the samples [61].

No matter the gas composition, results unequivocally demonstrate that PE is more beneficial than PP packaging. A key role is played by the gas permeability of the packaging material. The samples in PP packaging had significant moisture buildup, the package material collapsed, and the apples were highly contaminated. PE packages contained less moisture and seemed to preserve the gaseous atmosphere, as they did not collapse. The 88% CO_2/_12% N_2_ environment was where the most noticeable degradation of the PP-packaged apples was observed. The apples in the PE packaging showed very minimal fungal development, limited to the site of inoculation. A minor browning around the injection site was the only sign of degradation [61].

PP only prevented the synthesis of PAT in atmospheric gas and 58% CO_2/_42% N_2_, and it had no inhibitory effect on fungal growth in any of the investigated atmospheres. PE was highly effective and, depending on the modified environment, suppressed fungal growth by four- or fivefold. All three combinations of gases almost totally suppressed the development of PAT in apples packaged in PE. For all studied atmospheres, HPLC examination of the PE-packaged samples before and after the incubation time revealed that N_2_ levels rose and CO_2_ levels decreased. The research clearly showed that PE is a great way to store apples because it prevented *P. expansum* from growing, which led to the production of less than 3.2 µg/mL of PAT regardless of the atmospheric composition. PE reduced fungal growth by 68% and the production of the toxin by 99.5% without the need of a modified atmosphere [61].

In packinghouses, CA storage is typically utilized when apples need to be kept for longer than six months. Based on the mentioned studies, it appears that CA storage efficiency increases with storage duration. The most significant finding, though, is that CA storage appears to inhibit the accumulation of PAT in apples. Additionally, apples from CA storage appear to have substantially less PAT buildup following subsequent ambient storage than apples from traditional storage [47,48,60]. Thus, it appears that *P. expansum* may suffer irreversible harm from low O_2_ and high CO_2_ levels, which would impact PAT production both during CA storage and three days after subsequent ambient storage.

#### 2.1.3. Biocontrol Agents (BCAs)

Laws governing the use of pesticides have become increasingly stringent. Post-harvest fruit management is now based on complementary/alternative remedies that allow for the reduction in fungicide dosages or even the total avoidance of chemicals [62,63,64,65].

Because it is an environmentally safe approach, the use of antagonists—typically microorganisms isolated from fruit surfaces—that inhibit the growth of a particular pathogen is growing in popularity. These organisms are commonly referred to as BCAs. Their application is among the most promising substitutes for fungicides [62].

According to the review by Morales et al. [62], BCAs may cause a decline in the growth rate of the pathogen, but this could lead to an increase in secondary metabolism, more exactly the synthesis of PAT. This can be explained by the fact that mycotoxins are produced as a stress response, and BCAs might be perceived by the pathogen as stress factors [66]. However, in most cases, such as those presented below, BCAs are effective both in limiting the growth of *P. expansum* and in limiting the accumulation of PAT in the fruits.

Several in vitro assays [67,68,69] studied the influence of different microorganisms on PAT production. A *Rhodotorula glutinis* yeast strain showed significant growth when incubated together with *P. expansum.* In the same time, a significant decline in PAT buildup was observed. The metabolism of *R. glutinis* might be the cause of the reduction in PAT accumulation. The experiment was repeated in in vivo conditions as well. PAT synthesis was more diminished in treated apples than in apples that were not treated. According to the authors, biocontrol yeast cells in deteriorated tissues can metabolize PAT and adversely impact its accumulation [69].

The effectiveness of other antagonistic yeasts (*Metschnikowia pulcherrima* strain MACH1, *M. pulcherrima* strain GS9 and *M. fructicola* strain AL27) in the control of *P. expansum* growth and PAT buildup was studied as well. ‘*Granny Smith*’, ‘*Royal Gala*’, ‘*Golden Delicious*’ and ‘*Red Chief*’ apples were stored at 22 ± 1 °C for 7 days and at 1 ± 1 °C for 56 days. With the exception of ‘*Red Chief*’ cultivar, in which PAT content was remarkably higher in apples kept at low temperature, samples stored at 1 ± 1 °C for 56 days contained considerably lower PAT levels than those kept at room temperature. When compared to the control, the three antagonists were able more often than not to considerably lower the PAT content. At both temperatures, AL27strain proved to be the most effective BCA among all apple varieties, levels of PAT in ‘*Golden Delicious*’, ‘*Granny Smith*’ and ‘*Royal Gala*’ fruits treated with this BCA being similar to the chemical controls. This was especially true in case of cold storage, when PAT levels were lower in AL27-treated apples: 0.0 ng/g in ‘*Golden Delicious*’, 1.2 ng/g in ‘*Granny Smith*’, 24.0 ng/g in ‘*Royal Gala*’ (AL27 treatment) and 0.7 ng/g in ‘*Golden Delicious*’, 4.2 ng/g in ‘*Granny Smith*’, 29.5 ng/g in ‘*Royal Gala*’ (chemical controls). The fruits treated with GS9, the least effective antagonist, showed the highest quantities of PAT [70].

#### 2.1.4. Pathogen Load on the Fruits

*P. expansum*’s ability to synthesize PAT must also be considered from a relational, ecological point of view. Even if this fungus is psychrotrophic [71], synthesis of PAT molecules is the most efficient at 25 °C, the optimum temperature for *P. expansum*’s growth [72]. It must be admitted that this temperature is favorable for a large number of competing microorganisms, including opportunistic microbiota of apples, for which *P. expansum* has affinity.

By synthesizing PAT, *P. expansum* actually secures its competitive advantage to occupy a growth niche. Competition for resources might occur in intraspecific interactions [51].

Morales et al. [73] showed that competitor fungi can inhibit the growth and colonization by the PAT synthesizing fungus. Inoculation with competitors capable of rapid multiplication slowed down the rate of colonization of apples as early as the preharvest stage [74]. Remarkable results in this respect have been obtained using biopreparations of *Candida membranifaciens* and *Rhodotorula mucilaginosa* yeasts, which were able to inhibit *P. expansum* growth by 86% and reduce lesions by 96% [75].

### 2.2. Intrinsic Components That Influence PAT Production

#### 2.2.1. Susceptibility of Different Cultivars

Different apple varieties exhibit various susceptibilities to infection with *P. expansum* and toxin accumulation. A high number of apple cultivars were studied regarding their PAT production potential, and a high variability was observed.

There are certain cultivars, such as ‘*Golden Delicious*’, that are widely known for their predisposition to high PAT accumulation [76,77,78,79]. Several authors indicated that, in comparison with ‘*Fuji*’ variety, ‘*Golden Delicious*’ always accumulates more PAT [77,78,79,80]. They attribute it to the softer flesh and higher acidity of ‘*Golden*’ apples, which are some of the major factors that have impact on the amount of produced PAT.

Differences between cultivars were observed in several studies. Following the artificial inoculation with the same concentration of *P. expansum* suspension, ‘*Empire*’ and ‘*Red Delicious*’ cultivars showed no detectable PAT production, while ‘*McIntosh*’ and ‘*Golden Supreme*’ varieties produced the highest amounts of PAT (52.131 ppb and 54.221 ppb, respectively) [46]. Menniti et al. [76] found a more than three-fold higher PAT quantity in ‘*Golden Delicious*’ than in ‘*Royal Gala*’ (386 µg/kg and 114 µg/kg, respectively); they attributed it to the difference in acidity of the two varieties. ‘*Granny Smith*’ and ‘*Red Delicious*’ proved to be more resistant to soft rot, regardless the temperature of the environment, than ‘*Golden Delicious*’ and ‘*Fuji*’ varieties [77].

Recently, Kumar et al. [81] conducted an extensive study on the influence of intrinsic factors on *pat* genes, that are required for the synthesis of PAT and on *laeA* expression, which is a regulator of secondary metabolism [82]. As far as the cultivar’s effects on *laeA* and the PAT biosynthetic gene cluster, it was discovered that the interactions between sugar content, organic acid composition (malic acid especially), pH and phenolic compounds, which are specific for every apple variety, regulate fungal gene expression and PAT biosynthesis.

It can be concluded that there are differences in the susceptibility of different apple varieties to infection with *P. expansum* and, hence, to PAT production, but this element cannot be discussed without taking into account environmental factors, the microbial load on the surface of the fruit, the integrity of the apples, their biochemical and chemical content and the length of time and circumstances under which they have been stored. Physicochemical properties of the fruits, which have a very important role in vulnerability to PAT, are also variety-dependent to some extent.

#### 2.2.2. Physicochemical Properties of Apples

Fresh apple fruits have a water activity (a_w_) between 0.97 and 0.99 [83], which means that they fit within the minimum a_w_ limits for spore germination (0.83–0.85) and PAT synthesis (0.99) by *P. expansum* [44]. Old studies report a minimal water activity for PAT production of 0.95 [84,85].

Tannous et al. [83] obtained the biggest growth rate of *P. expansum* (0.6 cm/day), in vitro, at a_w_ of 0.99. At this value, PAT production was stimulated significantly, while at 0.95 a_w_ only traces of PAT were identified. When a_w_ was decreased to 0.85, a serious decrease in the growth was observed, and no PAT was produced. This is in accordance with the above mentioned studies regarding minimal a_w_ values for PAT production.

Besides a_w_, *P. expansum* growth, sporulation and PAT synthesis are all greatly impacted by ambient pH as well [86]. After harvesting of the fruits, changes in the pH appear, which impact the pathogenicity and physiology of *P. expansum*, which further regulates PAT synthesis in apples [35,78,86]. Two pH modulators, ammonia and D-gluconic acid, are released when infection with *P. expansum* occurs. They are considered precursors for PAT synthesis [87]. Ammonia modulates the activation of *PacC* [87]. *PacC* is activated under acidic environments and has a role in the modulation of pH response factors. Moreover, virulence, mycelial development and PAT production of *P. expansum* depend on *PacC* [88,89]. This proves once again the importance of pH in PAT synthesis.

*P. expansum* colonization and synthesis of PAT take place within a pH range of 2.5–6, and because apples generally have a pH between 3.1 and 4.2, they are considered to be a good environment for these processes [83,86]. McCallum et al. suggest that at pH below 3.5 PAT has a higher stability [54]. Zong et al. [86] discovered that a pH range between 3 and 5 was the most favorable for PAT production. In contrast, an increased pH negatively affects PAT production [77,90]. The cause of this may be the presence of the *pepg1* gene in the fungal genome, which is expressed only in acidic pH (maximum expression at pH 4, minimum at pH higher than 5). Therefore, pH influences the genes encoding PAT production [91].

Morales et al. [60] observed a general increase in PAT production in the pH range 2.5–3.5 of apple juices, but PAT levels did not change when pH increased to 5.5. Regarding the pH of the fruits, in ‘*Fuji*’ apples it was 4.26 and in ‘*Golden*’ 4.0. Fruit pH appeared to be a determining factor in PAT synthesis only during cold storage (‘*Golden*’ apples presented the lowest pH and accumulated higher PAT levels at 1 °C). This pattern was not seen at other temperatures, though, which leads to the conclusion that although pH does have an influence on PAT production, organic acid content also plays an important role.

Flesh firmness is another characteristic that influences to a large extent apple fruit’s resistance to blue mold and PAT accumulation, especially in domesticated cultivars [35]. Softening of the flesh during the ripening period automatically leads to a greater susceptibility to infection with various pathogens [92]. For this reason, in general, a negative correlation exists between flesh firmness and soft rot severity in apples, as well as PAT level [78,93,94]. Pearson correlation showed in a study by Konstantinou et al. [77] that vulnerability to *P. expansum* as well as PAT production of some commercial apple cultivars was negatively correlated to flesh firmness (r = 0.73 and 0.93, respectively).

#### 2.2.3. Chemical Composition of Apples

The amounts of ethylene, sugars and polyphenols are the most significant chemical components linked to the buildup of PAT in apples [35].

Ethylene is recognized as a key hormone that controls the ripening and softening of fruits and contributes to the resistance to pathogen attack. The main cause of late-ripening varieties’ increased resistance to degradation by soft rot has been identified as a lower rate of ethylene emission [95]. Additionally, apple’s natural ethylene production can serve as an indicator of the rate of wound healing [17,96]. Apples with faster wound healing capacity suffer a lower PAT buildup. The majority of apple varieties see an increase rather than a steady level in ethylene during the ripening process [97].

Likewise, a large number of polyphenols found in apples contribute to the defense against PAT contamination by scavenging the free radicals that PAT causes. Flavonols, procyanidins B1 and B2, epicatechin were found to be important for blue mold resistance [27,98]. Higher PAT content and higher gallic acid (r = 0.4226, *p* = 0.002), catechin (r = 0.3717, *p* = 0.008) and epicatechin (r = 0.3305, *p* = 0.019) contents are correlated positively [99]. These results were correlated because flavan-3-ols have a pro-oxidative effect that causes reactive oxygen species (ROS) to accumulate in the cells of *P. expansum*. This triggers the cellular antioxidant defense system and causes the production of PAT, which, as a secondary defense mechanism, reduces ROS levels in the cells [100,101].

In vitro study showed that quercetin and umbelliferone, two phenolic substances, are useful in lowering PAT accumulation [102].

Sugars, especially sucrose, are another important component of apples that regulate the buildup of PAT. In a culture media containing maltose, sucrose, and glucose as the only carbon source (at a final concentration of 10 g/L, which is about 29.2 mM), *P. expansum* strains T01, M1, and Pe21 had the maximum PAT accumulation (over 60 μg/mL) [86]. However, when sucrose content was increased from 15 to 175 mM, a decrease by 95% in PAT accumulation was observed [81,87,103]. Since a significant portion of the carbohydrates in apple fruits is sucrose, variations in sucrose content during fruit ripening may be linked to the synthesis of mycotoxin and fungal metabolism [35].

#### 2.2.4. Genetics

Recently, many advances have been made in the genetic research of *P. expansum*, including the identification of genes such as *patE*, *patG*, *patK*, *patL*, *patH* and *patI* that are involved in PAT synthesis [29,104,105,106]. Progress has also been made in the knowledge about *laeA*, the global regulator of secondary metabolism [103].

The genetic profile of different varieties is among the elements that influence the apples’ characteristics, controlling not only their capacity to heal bruises but also their susceptibility to *P. expansum* and, in turn, to PAT accumulation [107]. Zhong et al. reinforce this, as they mention that specific genetic information strongly regulates the defensive mechanism of wounded apples, determining whether they are resistant or vulnerable to the attack of *P. expansum* [35].

Apple varieties with close genetic make-up react to disease attacks in a similar way [108,109]. ‘*Golden Delicious*’ and its successors, ‘*Ariane*’ and ‘*Pink Lady*’ apples, showed a close pattern of growth of *P. expansum*, when the same quantity of spore suspension was used for their inoculation [108].

The *idh* gene is responsible for the effect of temperature and atmosphere on PAT synthesis. In vitro study demonstrated that at low temperatures, the expression of this gene is decreased, which lowers the pace of PAT production. Oxygen concentration in the atmosphere also affects PAT production by influencing the *idh* gene’s transcriptional level. When inoculated plates were kept in a controlled environment, at low temperature, a lower PAT accumulation was observed, as well as a down-regulation of the gene [110]. These finding support the results of Baert et al.’s study [53], that the combination of various stressors, such as low oxygen levels and cold temperatures lead to a decreased PAT production.

#### 2.2.5. Size of the Decay Area

Decay area is another cause of PAT contamination. PAT concentration generally increases with the increase in the decay rate. Garcia et al. [56] revealed that most isolates’ PAT production was strongly impacted by colony diameter, as colonies with a diameter of 60 mm accumulated more PAT than those with a 20 or 40 mm diameter.

A positive linear correlation was initially detected between the surface of the lesion and the concentration of PAT in apples, in Baert et al.’s study [53]. As the size of the rot grew, the PAT content also increased. However, afterwards, a decrease in PAT accumulation was observed. This reduction in PAT level has previously been reported [111] and is probably caused by the presence of enzymes that break down patulin.

Morales et al. [48,51] and Reddy et al. [50] have concluded that apparently the period of cold storage has a greater influence on the accumulation of PAT at a set lesion diameter than the lesion size itself. Still, the larger the initial lesions, the greater the PAT buildup was. For the artificial contamination of ‘*Golden Delicious*’ apples, a strong PAT-producer *P. expansum* strain was used in a study by Coton et al. They demonstrated that PAT content was significantly greater in surface samples (1 cm in depth) than in deeper samples, and within the lesions, with levels gradually decreasing in the surrounding tissues. It is advisable to remove at least 1 cm of tissue near and below a lesion sized up to 3 cm, in order to decrease PAT exposure [112]. This advice is consistent with Morales et al. [51], who found no PAT in the adjacent tissues (1 cm around a 2.5 cm diameter tissue).

#### 2.2.6. Toxin-Producing Capacity of the *P. expansum* Strain

Multiple studies have shown that the accumulation of PAT in apples is affected by the ability of the *P. expansum* strain to produce the toxin.

Six isolates obtained from Ontario, Canada, showed substantial variation regarding their growth and final dry weight of the mycelium. Based on this criterion, they were categorized into aggressively growing strains (PRD-1, P99418, PGD-1 and P18-5B,) and less aggressive strains (PM1 and P51-16B). Isolates’ levels of PAT synthesis varied: some produced no PAT at all (PM1, P51-16B), while others did produce more than 60 mg/g dry mycelial mass at 144 h incubation time at 25 °C (P99418-64.9 mg/g dry weight, P18-5B-66.6 mg/g dry weight). PRD-1 and PGD-1 strains produced 21.3 and 31.9 mg PAT/g dry weight, respectively. By producing secondary metabolites, such as PAT, the metabolism of fungi acidifies the growth medium, bringing the pH down to 3.5. PAT is the most stable at this value. The authors also discovered that every isolate had a distinct pH threshold value above which no PAT synthesis was observed. Remarkably, these values were within the pH range of a typical habitat for *P. expansum*, namely apple cider [54]. This is in accordance with another experiment, where the strains with the most aggressive growth (I12 and C28) produced the highest amounts of PAT, both in vitro and in vivo. In contrast, the least aggressive strain (I1) produced PAT in only a few culture media and cultivars [113].

Besides the contribution that the aggressivity of the strain has, the provenience of it is also of interest. The in vitro PAT producing potential of twenty-nine *P. expansum* isolates was verified during a study in Italy. All of them were isolated from pome fruits (apples and pears). In general, strains originating from yellow apples produced the highest levels of PAT (817.31 µg/cm^2^), succeeded by isolates from pears (2.73 µg/cm^2^) and red apples (2.21 µg/cm^2^). A variation in the PAT-producing capacity on a genetic level of the isolates (in the transcript level of a singular or several PAT biosynthetic genes) may be the cause of the varying levels of PAT production among the isolates, in vitro [114]. Isolates of *P. expansum* from Belgian apples and reference strains were tested for their PAT-producing capacity compared to the reference strains coming from apples and grapes, the isolates obtained from conventional and organic apples produced noticeably more PAT. The MUCL 29,189 reference strain had the lowest average PAT concentration, whereas FC116 isolate had the highest. The average concentrations of PAT ranged between 15 ± 2 and 747 ± 98 mg/kg [58]. This study demonstrated that PAT accumulation is mainly influenced by the *P. expansum* strain. In another study, the conclusions demonstrated the same correlation. Apart from a single one (P7), all other *P. expansum* strains (CadrP28af, PE97.IT, P6 and P37) caused massive infection in inoculated ‘*Golden Delicious*’ apples. Infected areas were 10–12 mm in diameter in the case of CadrP28af, PE97.IT, P6 and P3 strains, and 2 mm in diameter in the case of apples inoculated with P7. PAT levels ranged from below the detection limit up to 662 µg/kg. Four strains were able to produce PAT, but P6 did not produce the mycotoxin. Linear regression analysis showed that PAT production in this case could not be correlated with the incidence of infection (r = 0.47), nor with the severity (r = 0.39) and percentage (r = 0.53) of moldy area, but was strain dependent [76].

To reproduce in vitro the long-term storage conditions of apples, five isolates and one reference strain were selected from the total number of strains included in the study. The variability observed in PAT production under 25 °C and under long-term storage circumstances (25 °C for seven days, 1 °C for two months, 20 °C for three days) was different for the different isolates. Similar findings were reported by Garcia et al. [56]; however, Reddy et al. [50] reported a comparable variation in PAT synthesis across strains tested at various temperatures. These discrepancies show that when examining the impact of particular circumstances on PAT synthesis, a large number of isolates must be included. The influence of different temperatures on PAT production by different fungal strains was studied by others as well. Baert et al. [115] found that 83% of the *P. expansum* strains (from a total of 6) synthesize PAT at 1 °C, but at 4–7 °C and 10–12 °C, 95% and 100% accumulate PAT.

#### 2.2.7. Ripening Degree

Fruit ripening is known to have a significant role in the susceptibility of apples to diseases, as resistance to fungal infections during storage is influenced by the stage of fruit development at the time of harvest [116,117]. It has been demonstrated that more ripened fruits have a fairly higher PAT amount [81].

Several studies showed that apples with commercial maturity are less vulnerable to infection with *P. expansum* than overripe fruits [81,94,118,119,120]. It is the biochemical and physical changes that take place throughout the maturing process in apples that explain this phenomenon.

An overripe fruit is characterized by a high sugar content and reduced flesh firmness, ideal conditions for pathogen penetration and growth [23,27,35,94].

The loss of firmness has been linked to the solubilization of pectins via the arranged action of enzymes than modify cell wall structure (for example beta-galactosidase and beta-galactosidase 3) [121,122]. A high respiratory rate, along with high levels of ethylene release during storage, cause faster ripening, which eventually leads to the softening of the flesh [109,123,124], which favors fungal infection.

## 3. Conclusions

Apples’ patulin contamination, for which *P. expansum* is mostly responsible, is still a serious concern for public health and food safety. In this review, the interactions between intrinsic (apple variety, severity of decay area, toxin-producing capacity of the fungal strain, water activity, pH, flesh firmness, chemical composition of the fruits, genetics, pathogen load on the fruits, ripening degree) and extrinsic (storage temperature, composition of the air, packaging material, treatments with biocontrol agents) factors that affect fungal development and patulin accumulation were highlighted. Knowing these factors not only helps to clarify the dynamics of the fungal species’ physiology and pathogenicity, but it also lays the groundwork for creating focused control methods.

## Data Availability

In this literature-based review no new data were created. Previously published data and studies cited in the text are available in the public domain.

## Abbreviations

The following abbreviations are used in this manuscript:

PAT	Patulin
CA	Controlled atmosphere
PP	Polypropylene
PE	Polyethylene
HPLC	High-performance liquid chromatography
BCA	Biocontrol agent
a_w_	Water activity
ROS	Reactive oxygen species
